# Targeted Chinese Medicine Delivery by A New Family of Biodegradable Pseudo-Protein Nanoparticles for Treating Triple-Negative Breast Cancer: *In Vitro* and *In Vivo* Study

**DOI:** 10.3389/fonc.2020.600298

**Published:** 2021-01-20

**Authors:** Hiu Yee Kwan, Qinghua Xu, Ruihong Gong, Zhaoxiang Bian, Chih-Chang Chu

**Affiliations:** ^1^ School of Chinese Medicine, Hong Kong Baptist University, Hong Kong, China; ^2^ Biomedical Engineering Field, and Fiber Science Program, Department of Fiber Science and Apparel Design, Cornell University, Ithaca, NY, United States

**Keywords:** triple-negative breast cancer, gambogic acid, nanoparticle, poly(ester amide)s, apoptosis, tumor-associated macrophage

## Abstract

Triple negative breast cancer (TNBC) has the worst overall survival among all breast cancer subtypes; 80% of TNBC harbors TP53 mutation. Gambogic acid (GA) is an herbal compound isolated from the dry brownish gamboge resin of *Garcinia hanburyi*. A new family of biodegradable polymer, the folate (FA)-conjugated arginine-based poly(ester urea urethane)s nanoparticles (FA-Arg-PEUU NP), was developed as nano-carrier for GA. Its anti-TNBC effects and the underlying mechanism of action were examined. The average diameters of FA-Arg-PEUU NP and GA-loaded FA-Arg-PEUU NP (NP-GA) in water are around 165 and 220nm, respectively. Rhodamine-tagged FA-Arg-PEUU NP shows that the conjugation of FA onto Arg-PEUU NPs facilitates the internalization of FA-Arg-PEUU-NP into TNBC. Compared to free-GA at the same GA concentrations, NP-GA exhibits higher cytotoxicity in both TP53-mutated and non-TP53 expressed TNBC cells by increasing intrinsic and extrinsic apoptosis. In HCC1806-bearing xenograft mouse model, the targeted delivery of GA by the FA-Arg-PEUU-NP nano-carriers to the tumor sites results in a more potent anti-TNBC effect and lower toxicity towards normal tissues and organs when compared to free GA. Furthermore, NP-GA also reduces the tumor-associated macrophage (TAM) M1/M2 ratio, suggesting that the use of Arg-based nanoparticles as carriers for GA not only makes the surface of the nanoparticles positively charged, but also confers on to the nanoparticles an ability to modulate TAM polarization. Our data clearly demonstrate that NP-GA exhibits potent anti-TNBC effects with reduced off-target toxicity, which represents novel alternative targeted therapeutics for TNBC treatment.

## Introduction

Triple negative breast cancer (TNBC) is negative for estrogen receptor, progesterone receptor, and human epidermal growth factor receptor, which accounts for 10-17% of all breast cancer cases (1). TNBC has a distinct metastatic pattern. It is a high-grade invasive ductal carcinoma (2) and often spreads to the brain and lungs (3). TNBC is characterized by its poor prognosis and aggressive biological behavior. In a cohort of 1601 women with breast cancer, women with TNBC had an increased likelihood of death (hazard ratio 3.2; 95% CI, 2.3-4.5; p<0.001) within 5 years of diagnosis compared with other breast cancer subtypes. Indeed, TNBC is associated with the worst breast cancer specific and overall survival rates (hazard ratio for BCSS 2.99, 95% CI 2.59-3.45, p<0.001; hazard ratio for OS 2.72, 95% CI 2.39-3.10; p< 0.0001) among all breast cancer subtypes (3, 4).

TNBC patients have limited therapeutics options because they do not benefit from traditional anti-hormonal or anti-HER2-based therapies. The treatment approaches to TNBC include surgery, radiation therapy, and chemotherapy. Several targeted therapies are still under clinical trials. For example, neoadjuvant chemotherapy is used for TNBC patients with locally advanced disease, and this approach shows a high disease-free survival rate in TNBC patients (5) with 20% of the patients presenting a pathologic complete response (pCR) after neoadjuvant chemotherapy (6). However, TNBC patients who do not achieve pCR are very likely to suffer from early recurrence and die from metastatic diseases. Immunotherapy for TNBC is still under clinical trials (2, 7).

The Cancer Genome Atlas (TCGA) project has characterized the molecular profile of TNBC, including the mutations of the gene TP53. Up to 80% of TNBC harbors TP53 mutation (8). Protein p53 is one of the proteins that respond to DNA damage. Wild-type p53 protein induces the expressions of BCL-2 proteins, such as BIM, PUMA, and NOXA, that mediate the p53-dependent apoptosis; p53 mutant, however, cannot induce apoptosis in response to DNA damage in TNBC. Nevertheless, clinical data suggest that TNBC is sensitive to DNA-damaging agents such as the platinum-based drugs in chemotherapy (9), indicating that the induction of p53-independent apoptosis in TNBC is a promising therapeutic approach.

Gambogic acid (GA) is a xanthone structure isolated from the dry, brownish gamboge resin of *Garcinia hanburyi*. GA is a potent apoptosis inducer, as revealed by its structure-activity relationship (10). Studies show that GA triggers DNA damage signaling in liver cancer and non-small cell lung cancer (11). It also triggers the intrinsic caspase-dependent signaling pathway in neuroblastoma cells (12). In non-TNBC breast cancer, GA induces apoptosis by increasing reactive oxygen species (13); it also increases the sensitivity of breast cancer to TRAIL (TNF-related apoptosis-inducing ligand)-induced apoptosis by promoting the crosstalk between extrinsic and intrinsic apoptotic signaling pathways (14). Recently, a study also showed that GA increases sensitivity to paclitaxel in drug−resistant TNBC *via* the sonic hedgehog signaling pathway (15). He et al. also shows that GA-loaded biodegradable amino acid-based pseudo-protein nanoparticles exhibitspotent anticancer effects over free GA in several *in vitro* models, such as cervical cancer cells (Hela) and colorectal cancer cells (HCT116) (16).

Since GA is a potent anti-cancer agent, a phase IIa clinical trial has been conducted to test the efficacy of GA in treating non-small cell lung cancer (NSCLC), stomach cancer, colon cancer, breast cancer (non-TNBC type), liver cancer, and kidney cancer. Among these, GA enhanced the partial response (PR) in NSCLC, colon cancer, and renal cell cancer patients; GA also has a favorable safety profile at a dosage of 45mg m^-2^ (17). A follow-up clinical trial in phase IIb investigated the therapeutic effects of GA in NSCLC, renal cell cancer, and colon cancer; a phase III trial also investigated the effects in NSCLC and renal cell cancer. However, both trials showed that GA did not have an apparent advantage over chemotherapy in treating these cancers (Study report 138997640-2008ZX09101024/01 by Zhangleilei Jiangsu Kanion Pharmaceutical Company and Zhaoyiwu Jiangsu Kanion Pharmaceutical Company). Besides, GA has poor water solubility (<1µg ml^-1^) and it has a rapid clearance in plasma, which hinders its clinical applications. Therefore, a strategy to enhance the therapeutic effect of GA is needed.

Amino acid-based poly(ester amide)s (AA-PEAs) and their distant relative, poly(ester urea urethane) (AA-PEUU), are two relatively new families of biodegradable and biologically active polymers which have wide applications in the biomedical field (18–28). AA-PEAs are synthesized from three building blocks (amino acids, diols, and diacids), and AA-PEUU are prepared from four building blocks: amino acid, diols, diisocyanacide, and glycerol monoallyl ether. Amino acids such as arginine, lysine, and phenylalanine have been used to prepare AA-PEAs and AA-PEUUs with specific bioactivity. A series of AA-PEAs and AA-PEUUs with different structures have been developed in recent years to meet specific needs, ranging from wound dressings for burn treatments, drug eluting stent coatings for drug eluting stents and sutures, tissue engineering scaffolds, synthetic vaccines, and as drug and gene delivery carriers (23–28).

In a previously published *in vitro* study, He et al. designed a linear and branched Arg-based poly(ester urea urethane)s (Arg-PEUUs), folate (FA)-conjugated Arg PEUUs (FA-Arg-PEUUs), to deliver GA to different cancer cells like HeLa, A549, and HCT116 (16). The branched FA-Arg-PEUU NP design could improve the GA loading content and FA concentration on the surface of the nanoparticle compared to the linear FA-Arg-PEUU NP. When compared with free GA, the GA carried by the branched FA-Arg-PEUU NP exhibits higher cytotoxicity and induces more apoptosis and mitochondrial membrane potential disruption in the folate receptor-expressed cervical cancer cells and colorectal cancer cells (16).

In this study, we aimed to investigate the anti-TNBC effects of GA with a targeted delivery system utilizing folate (FA)-conjugated branched Arg-based PEUU biomaterial nano-carrier. FA and folate receptor alpha (FRα) are critical in regulating cell growth, DNA biosynthesis, repair, and methylation (29). FRα is expressed in 86% of the TNBC patients (30), which serves as an ideal marker for targeted delivery. The efficacy of the GA-loaded FA-Arg-PEUU NPs in TNBC was systematically examined by both *in vitro* and *in vivo* studies, and the underlining mechanism of action was elucidated to explain its potent anti-TNBC effects and the reduced off-target toxicity in comparison to free GA.

## Methods

### Materials

L-Arginine hydrochloride was purchased from Chem-Impex INT’L INC, hexamethylene diisocyanate (HDI) from Millipore Corporation, poly(ethylene glycol) (PEG, MCO 3400) from Aldrich, HHS-Rhodamine from Thermo Scientific, and gambogic acid (GA) was from Broadpharm. The dialysis bag with a MWCO of 10kg/mol was purchased from SnakeSkin™. PEG3400-NH_2_ and Folate N-hydroxysuccinimidyl ester (FA-NHS) were synthesized and purified according to the published procedure (16).

### Synthesis of Folate Modified Arg-PEUU (FA-Arg-PEUU)

To prepare branched arginine-based poly(ester urea urethane) (Arg-PEUU), pentathriol (0.0102g) and stannous 2-ethyl-hexanoate catalyst (9μL) were dissolved in the mixture of 20mL DMSO and 4mL DMF. The solution was stirred at 4°C and HDI was added. After reacting in a nitrogen atmosphere at 4°C for 30min, the prepolymer solution was moved to an oil bath at 45°C. Glycerol a-monoallyl ether (GAE, 0.02g) and Arg-4-Cl diester (1.27g) were dissolved in 10mL DMSO and added to the prepolymer solution under stirring for the polymerization reaction. After reacting for 8h, PEG3400-NH_2_ was added to cap the end of the PEUU chains and reacted for another 12h. The crude product of Arg-PEUU was obtained by precipitation in excess THF and dried under vacuum. The product was dissolved in DMSO and dialyzed in DI water for three days. The water was changed three times each day and the purified Arg-PEUU-PEG (6-Arg-4-PEUU-PEG) was collected by lyophilization (yield: 55%). Folate (FA) was tagged to the end of PEG to prepare the FA-Arg-PEUU. 6-Arg-4-PEUU-PEG (0.9g) was dissolved in 10mL DMSO and triethylamine (TEA, 12μL) was added to the solution. FA-NHS (0.145g) dissolved in 10mL DMSO was added to the 6-Arg-4-PEUU-PEG solution under stirring. After reacting in the dark at room temperature for 48h, the reaction solution was dialyzed in NaHCO_3_ (pH 8) twice and DI water for two days. The yellowish product of FA-Arg-PEUU was obtained by lyophilization.

### Preparation of Rhodamine-Labelled FA-Arg-PEUU Nanoparticle

FA-Arg-PEUU (0.5g) was dissolved in 10mL DMSO and bubbled with nitrogen for 10min. Cystamine (0.03mg) was added to the solution and the mixture was stirred in an oil bath at 70°C under nitrogen atmosphere for 24h. Cystamine was conjugated to the side chain of the polymer *via* the thiolene reaction and FA-Arg-PEUU was modified with amine group. The reaction solution was dialyzed in DI water for two days and lyophilized. The product (0.2g) was dissolved in 5mL DMSO and NHS-Rhodamine (0.002g) was added under stirring. After reacting under dark conditions for 48h, the product was purified through dialysis. The rhodamine-tagged FA-Arg-PEUU was collected by lyophilization with a yield of 80%. The rhodamine-tagged Arg-PEUU was synthesized using the same method. To prepare the rhodamine-labelled FA-Arg-PEUU nanoparticle (NP), the rhodamine-tagged FA-Arg-PEUU (0.05g) was dissolved in 5mL DMSO and added into 20mL DI water dropwise under vigorous stirring. The mixture solution was stirred for 2h and then dialyzed in DI water for 24h. The rhodamine-tagged FA-Arg-PEUU NP was lyophilized and kept at 4°C.

### Preparation of GA-Loaded FA-Arg-PEUU NP

The GA-loaded FA-Arg PEUU NP was prepared using a dialysis method. FA-Arg-PEUU was dissolved in DMSO with the concentration of 5mg/mL and GA was added into the FA-Arg-PEUU solution with a feeding ratio of 15% w/w. After stirring for 1h, the solution was transferred to a dialysis bag and dialyzed against DI water for 48h. The water was changed every 6h. The FA-Arg-PEUU nanoparticle (NP) suspension formed in the dialysis bag. The FA-Arg-PEUU NP was lyophilized and stored at 4°C. The GA-loaded Arg-PEUU NP was prepared in the same way. The size and surface potential of the GA-loaded FA-Arg PEUU NP was measured by using a Malvern Zetasizer Nano. The morphology of the nanoparticle was observed by Transition Electron Microscope (TEM, FEI T12 Spirit TEM STEM). The GA loading content (LC%) was measured by using a UV–Vis spectrophotometer (PerkinElmer Lambada 35, Madison). The GA-loaded nanoparticle (1mg) was suspended in 5mL ethanol and stirred at 45°C for 2h and GA was extracted into the solvent. The solution was filtered, and the ethanol was evaporated. The residue was dissolved in 1mL DMSO and GA content was determined by the absorption at 360nm. The LC was calculated through the following equation:

LC=mass of loaded GA/total mass of GA loaded nanoparticle×100%

### Cell Culture

Human triple negative breast cell lines HCC1806, HCC1143, and HCC1395 (ATCC) were maintained in RPMI-1640 medium and supplemented with 10% fetal bovine serum and 1% penicillin-streptomycin at 37°C incubator with 5% CO_2_ and 95% humidity.

### *In Vitro* Uptake of FA-Arg-PEUU NP

Uptake of FA-Arg-PEUU NP and Arg-PEUU NP by HCC1806 cells was observed by a Zeiss 710 confocal microscope and measured by flow cytometry (BD FACSAria Fusion). HCC1806 cells (1.0×10^5^/well) were incubated with rhodamine-tagged FA-Arg-PEUU NP or Arg-PEUU NP at a final concentration of 0.1mg/mL for 1h or 6h. The cells were washed with PBS and fixed with buffered formaldehyde (1.5mL, 4%, w/v) for 10 min; cell nuclei were stained with DAPI for 3 min. Cell images were taken by Zeiss 710 confocal microscope. In a flow cytometry study, cells were treated with the same protocol; the treatment period was 1h, 3h, or 14h. Fluorescence intensities of intracellular nanoparticles were analyzed by flow cytometer.

### MTT Assay

HCC1806, HCC1143, and HCC1395 cells (5000 cells/well) were incubated with free-GA or GA-loaded FA-Arg-PEUU NP (NP-GA) for 3h at 37°C before MTT assay was performed. Signal was read by spectrometer at 574nm. IC_50_ values (the GA concentration for the apoptosis study) were calculated using Graph Pad.

### Annexin V/PI Dye Staining

HCC1806, HCC1143, and HCC1395 (5×10^5^ cells/well) were treated by free-GA or NP-GA with the dosages of GA fixed at IC_50_ values (30.27µm, 14.51µm, and 15.76µm for HCC1143, HCC1806, and HCC1395 cells, respectively). After 3h-treatment, apoptotic cells were assessed using Annexin V-FITC apoptosis detection kit (BD bioscience).

### Western Blot Analysis

HCC1806, HCC1143, and HCC1395 (5×10^5^ cells/well) was treated by free-GA or NP-GA for 3h. Whole cell lysates were obtained by suspending the cells in lysis buffer followed by centrifugation; tumors were dispersed in lysis buffer by sonication before centrifugation, and supernatants were collected. Total protein concentrations in these samples were measured by Pierce BCA protein Assay. Proteins were separated on 10% SDS-PAGE and transferred onto polyvinylidene difluoride membranes. After blocking with 5% milk in TBST for 1h, the membrane was incubated with the respective primary antibody (cleaved caspase-3, -6, -7, -8, -12, and cleaved PARP) overnight at 4°C before incubating with the secondary antibody for 1h. Chemiluminescence signals were detected by enhancing ECL (Bio-Rad).

### Mitochondrial Membrane Potential

HCC1143, HCC1806, and HCC1395 cells were treated by free GA or NP-GA at its IC_50_ for 3h (30.27µm for HCC1143; 14.51µm for HCC1806, 15.76µm for HCC1395). Cells cultured in media served as controls. Changes in mitochondrial membrane potential were measured by cationic lipophilic dye JC-10 (Abcam) and the data were presented as the ratio of green fluorescent signal (520nm) to red fluorescent signal (590nm).

### HCC1806-Bearing Xenograft Mouse Model

All the animal studies obtained ethical approval from the Research Ethics Committee of the Hong Kong Baptist University. Female nude mice of 4-week old were obtained from The Chinese University of Hong Kong. Mice were kept in an animal room with 12h light/dark cycle and temperature control. Food and water were available *ad libitum*. HCC1806 (1×10^6^ cells/100μL) were suspended in PBS and inoculated into the left armpit of the mice. Tumors were formed 1 week after inoculation.

### Nanoparticle Carrier Tissue Distribution in HCC1806-Bearing Xenograft Mouse Model

Rhodamine-tagged Arg-PEUU NP or rhodamine-tagged FA-Arg-PEUU-NP at 72.7mg/kg was given to the TNBC-bearing xenograft mouse model by an intravenous route *via* the tail vein. Mice were killed at 30min, 1h, 2h, 4h, and 6h after injection. Tumors and major organs were collected. Images were acquired in IVIS Lumina XR imaging system with an exposure time of 1.5s (PerkinElmer).

### Anti-Tumor Activity of Free-GA or NP-GA

Mice were randomly divided into groups when tumors were grown to 100mm^3^. Free-GA or NP-GA at a dosage of 4mg kg^-1^ or 8mg kg^-1^ were given to mice *via* intravenous injection every two days for 17 days. Control groups were given vehicle or non-GA-loaded FA-Arg-PEUU NP. Body weight and tumor size were monitored every day. Tumor size was calculated by the formula *a* ^2^×*b*×0.4, where *a* is the smallest diameter and b is the diameter perpendicular to *a*.

### Tissue and Organ Toxicity Analyzed by H&E Staining

After mice were sacrificed, tumors, hearts, lungs, livers, kidneys, and spleens were dissected and fixed in 10% neutral buffered paraformaldehyde at 4°C for 24h. Tissue sections were stained by hematoxylin and eosin (H&E). Image pictures were taken by microscope (NIKON Eclipse) and analyzed by a pathologist.

### Immunohistochemistry

Paraffin sections of tumors were deparaffinized and rehydrated. Endogenous peroxidase was quenched by incubating with 3% H_2_O_2_ before blocking by serum. The sections were incubated with anti-cleaved caspase 3 or cleaved PARP at 4°C overnight before incubating with biotinylated secondary antibody for 30 min, peroxidase substrate for 10 min, and then being deionized in water for 5 min and counterstained with hematoxylin.

### Tumor Associated Macrophage (TAM) Isolation

Mice were anesthetized by isoflurane. Tumors were dissected and kept in a serum-free medium. Liberase DL solution (Roche), Liberase TL solution (Roche), and DNase I were added to the samples, mixed, and incubated for 45 min at 37°C under continuous shaking. PBS was used to terminate the enzymatic digestion. Tumor cells were filtered using a 100μm cell strainer, centrifuged, and suspended in 1% w/v BSA containing PBS. TAM was isolated from tumors using Anti-F4/80 Microbeads Ultrapure Kit (Miltenyi Biotec). TAM was incubated with anti-CD80 or CD206 antibody for 30 min before analysis with flow cytometry (BD Calibur). FlowJo software was used to analyze the data.

### Bio-Distribution of GA-Loaded FA-Arg-PEUU NP

Free-GA and NP-GA (4mg kg^-1^ or 8mg kg^-1^) were given to the TNBC-bearing xenograft mouse models by an intravenous route *via* the tail vein. Mice were killed 15min, 30min, 1h, 2h, and 4h after injection. Tumors, serum, and major organs were collected. Serum samples and homogenized tumors were extracted with 1mol/L HCl and ethyl acetate and reconstituted in mobile phase for LC/MS analysis which was performed by Agilent 6460 Triple-Quad Mass Spectrometer. Gradient chromatographic separation was performed on a Luna C18 column (Phenomenex) with a security C18 guard column (Phenomenex). Mobile phase was delivered at 1 ml/min; column temperature was maintained at 40°C.

### Statistical Analysis

All statistical analyses were performed using GraphPad Prism software (Version 5.00). The quantitative data were presented as mean ± standard error of mean (SEM).

## Results

### FA Conjugation Enhances the Internalization of Arg-PEUU NP Into TNBC Cells

**Figure 1:**
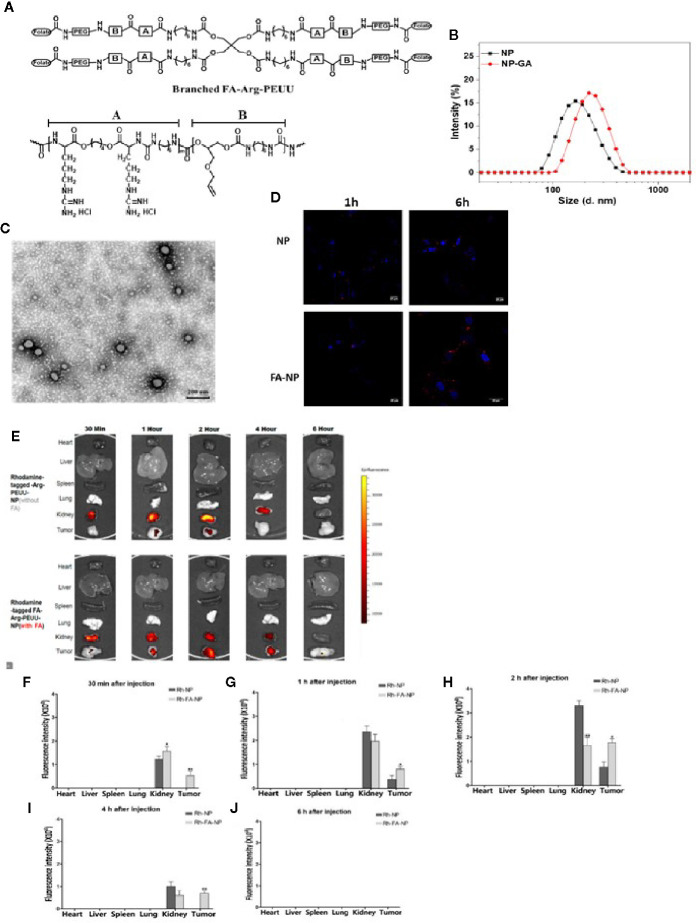
Uptake of the rhodamine-labelled FA-Arg-PEUU and Arg-PEUU nanoparticles by TNBC cells, and the tumor and major organs in the HCC1806-bearing xenograft mouse models. **(A)** The structure of branched FA-Arg-PEUU (6-Arg-4-PEUU), **(B)** size distribution of FA-Arg-PEUU NP and GA-loaded FA-Arg-PEUU NP (NP-GA), **(C)** transmission electron microscopy (TEM) image of NP-GA. **(D)** Confocal laser scanning microscopy images of HCC1806 cells after being incubated with rhodamine-labeled Arg-PEUU NP (Rh-NP) and FA-Arg-PEUU NP (Rh-FA-NP) for 1h and 6h. Red dots are rhodamine-labeled NPs. **(E)** The biodistribution and **(F–J)** fluorescent intensities of the rhodamine-tagged Arg-PEUU (Rh-NP) and rhodamine-labelled FA-Arg-PEUU nanoparticles (Rh-FA-NP) in the heart, liver, spleen, lung, kidney, and tumor of the HCC1806-bearing xenograft mouse model at the indicated time points after the injection of Rh-NP or Rh-FA-NP. The data are shown as the means ± SEM, n = 3 mice in each group, *p < 0.05, **p < 0.01.

FA-Arg-PEUU and GA-loaded FA-Arg-PEUU NP (NP-GA) were first prepared. The structure of the FA-Arg-PEUU is shown in **Figure 1A**. The branched polymer self-assembled into nanoparticles in aqueous solution. The average diameters of FA-Arg-PEUU NP and GA-loaded FA-Arg-PEUU NP (NP-GA) in water were around 165 and 220nm, respectively (**Figure 1B**). The surfaces of FA-Arg-PEUU NP and GA-loaded FA-Arg-PEUU NP were positively charged (25.3 and 26.8mV, respectively) due to the presence of the Arg component. The GA-loaded FA-Arg-PEUU NP had a spherical nano-micellar structure (**Figure 1C**). The GA loading content inside FA-Arg-PEUU NP was ~11%, which was 123.6µg GA per mg of FA-Arg-PEUU NP. In our previous *in vitro* release study (16), 40% GA was cumulatively released from the GA-loaded FA-Arg-PEUU NP at 18 hours, and 90% at 60 hours. We also examined the stability of the FA-Arg-PEUU NP. FA-Arg-PEUU-NP solution was stored at 4°C and the NP size was tested after 1 and 3 days. The Z-average diameter of the FA-Arg-PEUU NP was about 165 nm after 1 day and 156 nm after 3 days, indicating that the GA-loaded NP is relatively stable in aqueous solution.

Confocal imaging and flow cytometer techniques were used to examine whether the FA conjugation onto Arg-PEUU-NP enhances the internalization of the NPs into the TNBC cells (HCC1806) which express folate receptors (30). In rhodamine-labeled Arg-PEUU-NP (Rh-NP) and FA-Arg-PEUU-NP (Rh-FA-NP), the NPs are modified with rhodamine by a chemical bond in which rhodamine is a part of the polymer structure. Data show that both rhodamine-labeled Rh-NP and Rh-FA-NP could be taken up by HCC1806 cells as early as 1h (**Figure 1D**). More nanoparticles entered the cells as the incubation time increased from 1h to 6h. The much faster rate and higher level of endocytosis of the Rh-FA-NP compared to non-FA-tagged Rh-NP indicated the targeting effect of FA-modified nanoparticles toward HCC1806 cells.

The targeting effect of FA-tagged NP toward TNBC is further suggested by the subsequent *in vivo* tumor localization study with the HCC1806-bearing xenograft mouse model (**Figures 1E–J**). Thirty minutes after the injection, Rh-FA-NP started to accumulate at the tumor sites and its concentration kept increasing until 2h post-injection. In contrast, the non-FA tagged Rh-NP started to accumulate at tumor sites 1h post-injection. The non-FA tagged Rh-NP was totally cleared from the tumor site 4h after the injection, but it took 6h for the FA-tagged Rh-FA-NP to clear. Furthermore, the signals of Rh-FA-NP at tumor sites were significantly higher than Rh-NP in all the measurements (**Figures 1F–J**). Taken together, both the *in vitro* and *in vivo* data suggest that FA conjugation onto the Arg-PEUU NPs facilitates the internalization of FA-Arg-PEUU-NP into TNBC.

### NP-GA Exhibits Higher Cytotoxic Effect Than Free GA in TNBC Cells

**Figure 2:**
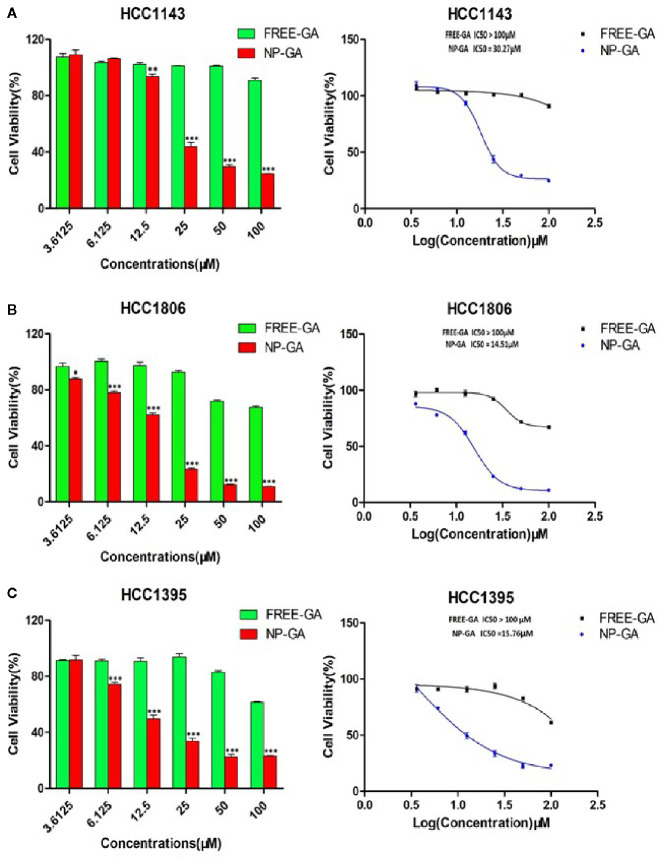
The *in vitro* cell viability of TNBC as a function of the GA drug and the NP carrier concentrations. **(A)** HCC1143, **(B)** HCC1806, and **(C)** HCC1395 after free-GA or GA-loaded FA-Arg-PEUU-NP (NP-GA) treatments for 3h at the indicated concentrations. X-axis on the left panel shows the concentrations of free-GA and NP-GA in µM; x-axis on the right panel shows the curves of the corresponding log concentration values of the free-GA and NP-GA. The data are shown as the means ± SEM, n = 3, *p < 0.05, **p < 0.01, ***p < 0.001 compared to free GA.

Three TNBC cell lines (HCC1143, HCC1806, and HCC1395) expressing folate receptors (30) were used for cell viability study. HCC1143 and HCC1395 harbor TP53 mutation, while HCC1806 has no p53 expression (31). It is worth noting that TP53 gene mutations are the dominant mutations in TNBC (32). **Figure 2** showed that GA delivered by FA-Arg-PEUU-NP carriers (NP-GA) had significantly higher cytotoxicity in all three TNBC cell lines when compared to free GA (after 3h-incubation). The IC_50_ for the NP-GA was 30.27µm for HCC1143 cells, 14.51µm for HCC1806 cells, and 15.76µm for HCC1395 cells, while the IC_50_ for free GA was over 100µm in all these cell lines. The viability of HCC1143 cells was 90.94% after free GA treatment, and was 24.64% after the NP-GA treatment at the same GA dosage of 100µm (**Figure 2A**). Similar cell viability trends were observed in the two other cell lines: HCC1806 cells were 67.40% and 11.08% after free GA and NP-GA treatments at the GA dosage of 100µm, respectively (**Figure 2B**). HCC1395 cells were 61.50% and 23.18% after free GA and NP-GA treatments at the GA dosage of 100µm, respectively (**Figure 2C**). However, the cytotoxicity of the free GA and NP-GA in the HCC1806 and HCC1143 cells were not significantly different after 24-h treatment (**Supplementary Figure S1**), which may due to the GA levels in the free-GA-treated cells and NP-GA-treated cells becoming similar after a long incubation period of 24h.

**Figure 3:**
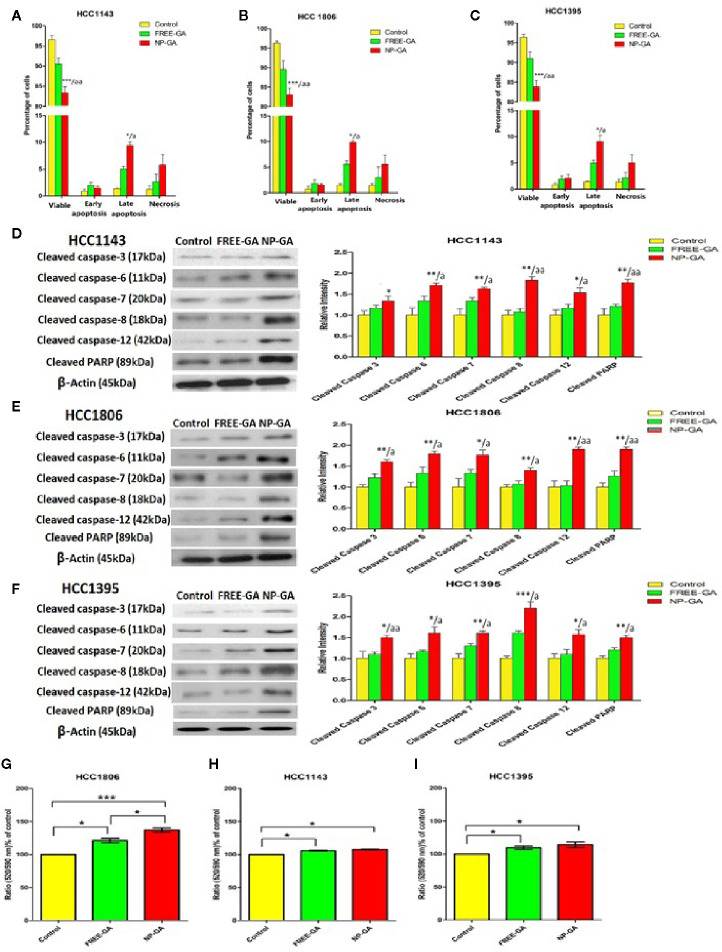
The percentages of the viable, early apoptotic, late apoptotic, and necrotic TNBC cells after free-GA and NP-GA treatments for 3 hours. Apoptosis and necrosis in **(A)** HCC1143, **(B)** HCC1806, and **(C)** HCC1395 cells after free-GA or GA-loaded FA-Arg-PEUU-NP (NP-GA) treatment for 3h. Cleaved caspase protein expressions in **(D)** HCC1143, **(E)** HCC1806, and **(F)** HCC1395 cells after free-GA or GA-loaded FA-Arg-PEUU-NP (NP-GA) treatment for 3h. Left panel: Western blot showing the protein expressions of cleaved caspase-3, cleaved caspase-6, cleaved caspase-7, cleaved caspase-8, cleaved caspase-12, and cleaved PARP. Right panel: the quantitative analysis of the corresponding protein expressions. The data are shown as the means ± SEM, n = 3, *p < 0.05, **p < 0.01, ***p < 0.001 compared to control group; a < 0.05, aa < 0.01 compared to free GA group. Changes in mitochondrial membrane potential (MMP) in **(G)** HCC1806, **(H)** HCC1143, and **(I)** HCC1395 cells after free-GA or GA-NP treatment for 3h. The y-axis is the ratio of green fluorescent signal (520nm) to red fluorescent signal (590nm). An increase in 520/590nm ratio indicates the collapse of MMP. The data are shown as the means ± SEM, n = 3, *p < 0.05, **p < 0.01, ***p < 0.001 as indicated.

Annexin V-FITC/PI staining was used to assess the percentage of the apoptotic cells after treatments. All three TNBC cell lines were treated with free GA or NP-GA for 3h and the dosages of GA were fixed at the IC_50_ values of NP-GA (30.27µm, 14.51µm, and 15.76µm for HCC1143, HCC1806, and HCC1395 cells, respectively). The NP-GA treatment significantly induced more apoptosis in these cell lines than the free GA treatment (**Figures 3A–C** and **Supplementary Figure S2**). The percentages of HCC1143 apoptotic cells were 7.04% and 10.90% after free GA and NP-GA treatments, respectively (**Figure 3A**). Similar apoptosis data were observed in the two other TNBC cell lines: the percentages of HCC1806 apoptotic cells were 7.48% and 11.44% after free GA and NP-GA treatments, respectively (**Figure 3B**). The percentage of HCC1395 apoptotic cells were 7.13% and 14.08% after free GA and NP-GA treatments, respectively (**Figure 3C**). However, NP-GA did not significantly affect the percentages of the necrotic cells in these cell lines (**Figures 3A–C**).

The *in vitro* apoptosis data in **Figures 3A–C** was further examined in terms of the apoptotic pathways. As shown in **Figures 3D–F**, NP-GA enhanced both intrinsic and extrinsic apoptotic pathways as indicated by the elevated levels of cleaved caspases 8 & 12, which led to the increased cleavage of caspases 3, 6, 7, and poly(ADP-ribose) polymerase (PARP). The data suggest that NP-GA exerts a higher apoptotic effect than free GA in TNBC cells regardless of the TP53 mutation status, which underlie the enhanced cytotoxicity of the NP-GA toward the TNBC cells.

Since collapse of mitochondrial membrane potential will initiate the apoptotic program, changes in the mitochondrial membrane potential (ΔΨm) of the TNBC cells were examined. ΔΨm was measured by cationic lipophilic dye JC-10 which forms reversible red-fluorescent aggregates in the mitochondria with a polarized mitochondrial membrane. When mitochondrial membrane potential collapses, the cells fail to retain JC-10 in the mitochondria and the dye returns to its monomeric green fluorescent form. Therefore, increase in the ratio of green fluorescent signal (520nm) to red fluorescent signal (590nm) indicates the collapse of mitochondrial membrane potential. Compared to control, both free-GA and NP-GA disrupted the mitochondrial membrane potential as indicated by the elevated 520/590nm ratio (**Figures 3G–I**). Free GA increased the 520/590nm ratio by 21.02%, 5.63%, and 9.55% in HCC1806, HCC1143, and HCC1395 cells, respectively. NP-GA increased the 520/590nm ratio by 36.94%, 7.55%, and 14.22% in HCC1806, HCC1143, and HCC1395 cells, respectively. A significant difference of the 520/590nm ratio between free GA and NP-GA treatments was observed in HCC1806 cells, in which NP-GA significantly increased the ratio by 15.92% (**Figure 3G**). However, there has no significant difference in HCC1143 and HCC1395 cells (**Figures 3H, I**).

Our data on apoptosis study suggest that NP-GA exerts a higher apoptotic effect than free GA in all three TNBC cell lines, which is not due to ΔΨm but instead to the caspase activation independent of the mitochondrial depolarization.

### NP-GA Has a More Significant Anti-TNBC Effect Than Free GA in HCC1806-Bearing Xenograft Mouse Model

**Figure 4:**
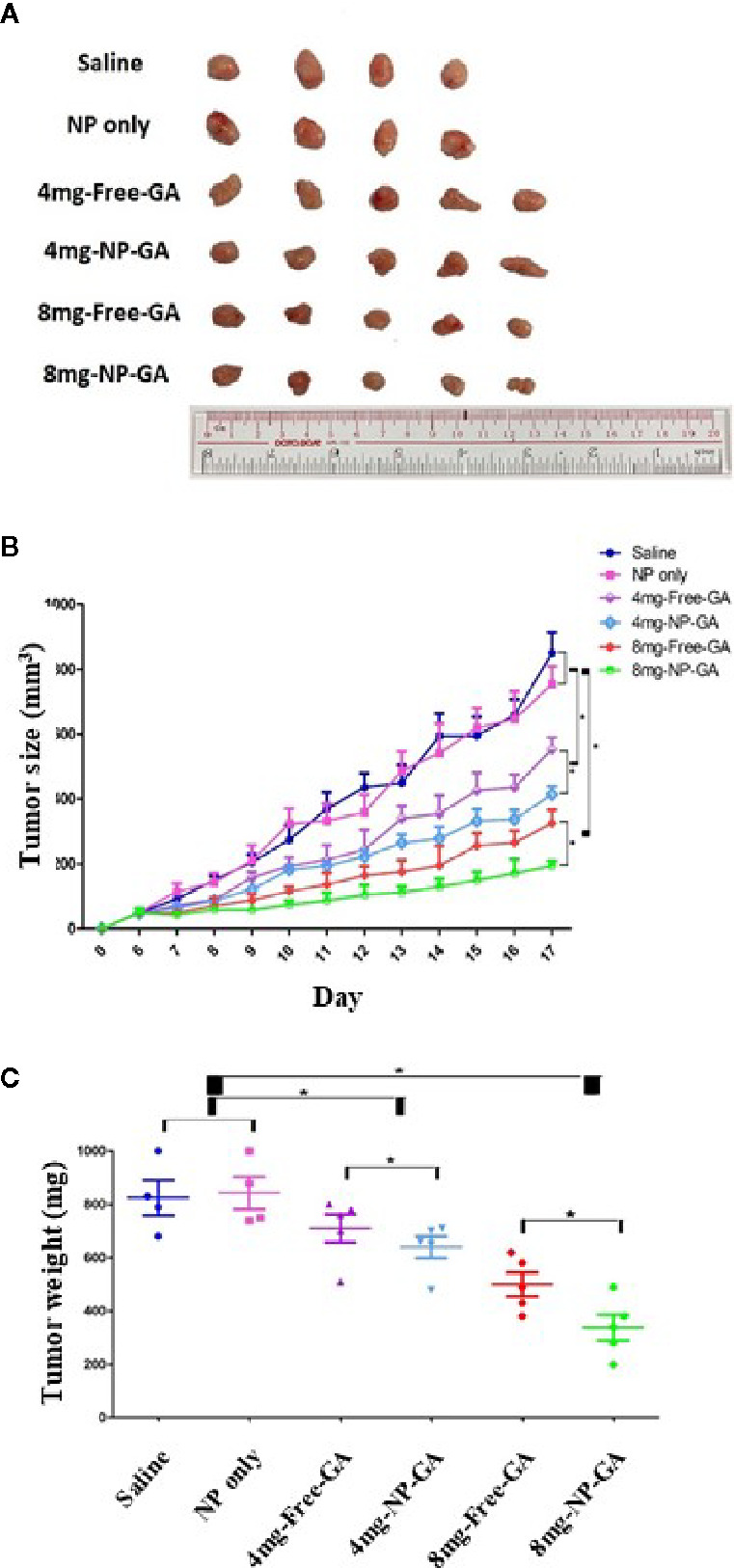
The tumor size and tumor weight of the HCC1806-bearing xenograft mouse models after saline, FA-Arg-PEUU-NP (NP), free GA, or GA-loaded FA-Arg-PEUU-NP (NP-GA) treatments. **(A)** Images, **(B)** volume, and **(C)** weight of the tumors dissected from the HCC1806-bearing xenograft mouse model after the saline, FA-Arg-PEUU-NP (NP), free GA, or GA-loaded FA-Arg-PEUU-NP (NP-GA) treatments. Day 0 indicated the day on which the tumor cells were inoculated into the nude mice. The data are shown as the means ± SEM, n = 4-5 mice in each group. *p < 0.05 as indicated.

The anti-TNBC effects were also examined in HCC1806-bearing xenograft mouse model. Mice were treated with free GA or NP-GA at 4 or 8mg kg^-1^ GA equivalents by intravenous injection when the tumors were grown to 100mm^3^. Saline and blank FA-Arg-PEUU-NP (NP) were used as controls. NP alone did not significantly affect the tumor size and tumor weight when compared to the saline control group (**Figures 4A–C**), suggesting NP *per se* does not affect the growth of TNBC in these mice. However, NP-GA was more potent in reducing the tumor size and tumor weight when compared to free GA at the same GA dosage (**Figures 4A–C**). After 17 days of treatment, the average tumor weights for the NP-GA groups were statistically less than the free GA groups at both 4 and 8mg kg^-1^ GA dosages (**Figure 4C**). The average tumor weight was 825mg for the saline group, 843mg for the NP group, 710mg and 640mg for the 4mg-free-GA group and 4mg-NP-GA group, respectively, and 500mg and 338mg for the 8mg-free-GA group and 8mg-NP-GA group, respectively.

**Figure 5:**
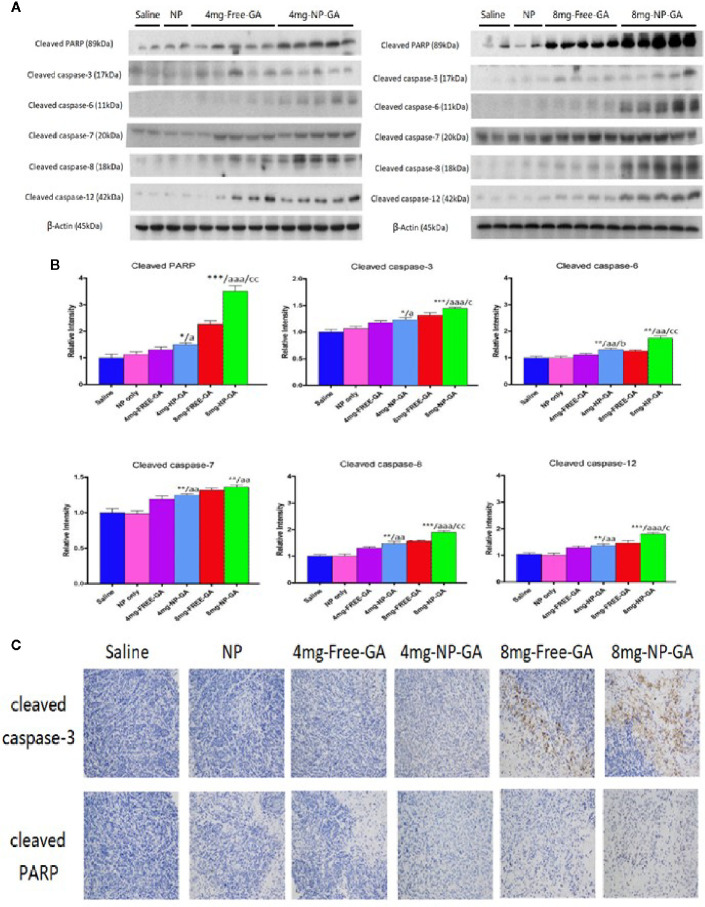
*In vivo* apoptotic protein expressions in the xenograft tissues of the HCC1806-bearing xenograft mouse models after saline, FA-Arg-PEUU-NP (NP), free GA, or GA-loaded FA-Arg-PEUU-NP (NP-GA) treatments. **(A)** Western blot showing the protein expressions of cleaved caspase-3, cleaved caspase-6, cleaved caspase-7, cleaved caspase-8, cleaved caspase-12, and cleaved PARP in the xenograft tissues and **(B)** quantitative analysis of the corresponding protein expressions. The data are shown as the means ± SEM, n = 4-5 mice in each group. *p < 0.05, **p < 0.01, ***p < 0.001 compared to saline group; a < 0.05, aa < 0.01, aaa < 0.001 compared to NP group; b < 0.05 compared to 4mg-free-GA group; c < 0.05, cc < 0.01 compared to 8mg-free-GA group. **(C)** Representative pictures showing the immunohistochemistry (IHC)**** staining of the cleaved capase-3 and cleaved PARP in the xenograft tissues in these mice.

The study of apoptotic markers in the tumor tissues suggest that NP-GA at both GA dosages induces more apoptosis in the tumors when compared to free GA, as shown by the elevated expression levels of cleaved caspases 12, 8, 3, 6, 7, and PARP in the tumor tissues (**Figures 5A, B**). These *in vivo* data were in parallel with the *in vitro* data (**Figures 3D–F**), which further suggest NP-GA induces more intrinsic and extrinsic apoptosis than free GA in TNBC. Immunohistochemistry study also suggests that the expressions of cleaved caspase 3 and PARP in the tumor tissues are higher in the NP-GA group than the free GA group (**Figure 5C**). The enhanced apoptosis in the tumors may underlie the potent anti-TNBC effects of the NP-GA treatments.

### The FA-Arg-PEUU-NP Carriers Increase Delivery of GA to TNBC In Vivo

**Figure 6:**
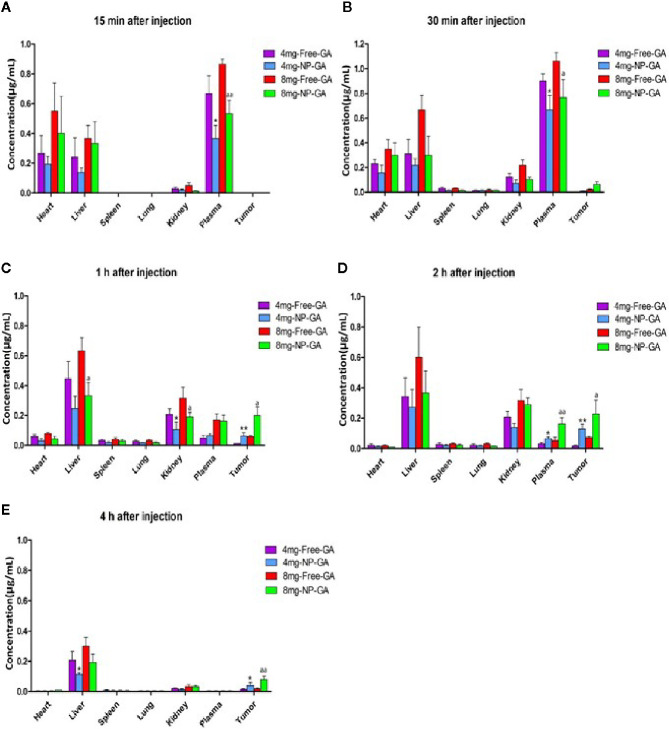
Biodistribution of GA as a function of time in a variety of tissues in the HCC1806-bearing xenograft mouse models after free GA or GA-loaded FA-Arg-PEUU-NP (NP-GA) treatments. **(A)** 15 min, **(B)** 30 min, **(C)** 1h, **(D)** 2h, and **(E)** 4h after intravenous injection of free GA or GA-loaded FA-Arg-PEUU-NP (NP-GA) at the indicated dosage. The data are shown as the means ± SEM, n = 3 mice in each group. *p < 0.05, **p < 0.01 compared to 4mg-free-GA group; a < 0.05, aa < 0.01 compared to 8mg-free-GA group.

**Figure 6** shows the LC-MS analysis of the GA levels in various organs and the tumors in the TNBC-bearing xenograft mouse models over a period of 4h after free GA or NP-GA treatments at the GA dosage of either 4 or 8mg kg^-1^. At the 8mg kg^-1^dosage, at 30 min post-administration, GA started to accumulate in the tumors of the free-GA and NP-GA groups (**Figure 6B**); the free GA group had 0.02µg mL^-1^ while the NP-GA group had 0.06µg mL^-1^ of GA in the tumors, i.e., 3 times more than the free GA group. A similar trend was also observed at 1h and 2h post-administration. At 1h post-administration, the free GA group had 0.06µg mL^-1^ while the NP-GA group had 0.20µg mL^-1^ of GA in the tumors (**Figure 6C**); at 2h post-administration, the free GA group had 0.07µg mL^-1^ while the NP-GA group has 0.23µg mL^-1^ in the tumors (**Figure 6D**). At the 4mg kg^-1^ dosage, the NP-GA treatment barely registered a GA level in the tumor. At 30-minute post-administration, no GA could be detected in the tumor from the free GA group. At 1h post-administration, the free GA group had 0.01µg mL^-1^ and NP-GA group had 0.06µg mL^-1^ of GA in the tumors (**Figure 6C**); at 2h post-administration, the free GA group had 0.02µg mL^-1^ and NP-GA group had 0.13µg mL^-1^ of GA in the tumors (**Figure 6D**).

Since NP-GA was administrated by an intravenous route *via* the tail vein, the NP-GA was detected in the blood circulation 15 minutes post-administration (**Figure 6A**). Interestingly, our data showed that at 2h post-injection (**Figure 6D**), the concentration of GA in the plasma was higher in the NP-GA groups than that in the free-GA groups, which were 0.03µg mL^-1^ GA in the free-GA group and 0.07µg mL^-1^ in the NP-GA group at 4mg kg^-1^ dosage. At the 8mg kg^-1^ dosage, it was 0.06µg mL^-1^ GA in the free-GA group and 0.16µg mL^-1^ in the NP-GA group. These data imply that GA carried by the NP carriers may have an increased circulating half-life.

GA was also detected in the liver because the liver functions to filter the blood. The concentrations of GA in the liver are lower in the NP-GA group when compared to free GA at all the time points. At 1h post-administration, at the 4mg kg^-1^ dosage, mice treated by free GA had 0.45µg mL^-1^ of GA in their livers, and those treated by NP-GA had only 0.25µg mL^-1^ of GA in their livers. At the 8mg kg^-1^ dosage, the free GA group had 0.63µg mL^-1^ and NP-GA group had 0.33µg mL^-1^ in their livers (**Figure 6C**). A similar trend was also observed at both 2h and 4h post-administration (**Figures 6D, E**).

NP-GA treatment also resulted in a lower GA concentration in the kidneys when compared to free GA at 30 minutes post-administration. At the 4mg kg^-1^ dosage, the free GA group had 0.12µg mL^-1^ and NP-GA group had only 0.07µg mL^-1^ of GA in the kidneys; at the 8mg kg^-1^ dosage, the free GA group had 0.22µg mL^-1^ and NP-GA group had only 0.10µg mL^-1^ in the kidneys (**Figure 6B**). At 1h post-administration, at 4 mg kg^-1^ dosage, the free GA group had 0.21µg mL^-1^ and NP-GA group had only 0.11µg mL^-1^ of GA in the kidneys; at 8 mg kg^-1^ dosage, the free GA group had 0.32µg mL^-1^ and NP-GA group had only 0.19µg mL^-1^ in the kidneys (**Figure 6C**). A similar trend was also observed at 2h post-administration (**Figure 6D**).

These results clearly demonstrated that mice treated by NP-GA had significantly higher GA levels in the tumors than those that received free-GA treatments, whereas the GA concentrations in the major organs were reduced, suggesting that NP-GA has fewer off-target side effects.

### NP-GA Has Lower Off-Target Side Effect Compared to Free GA

**Figure 7:**
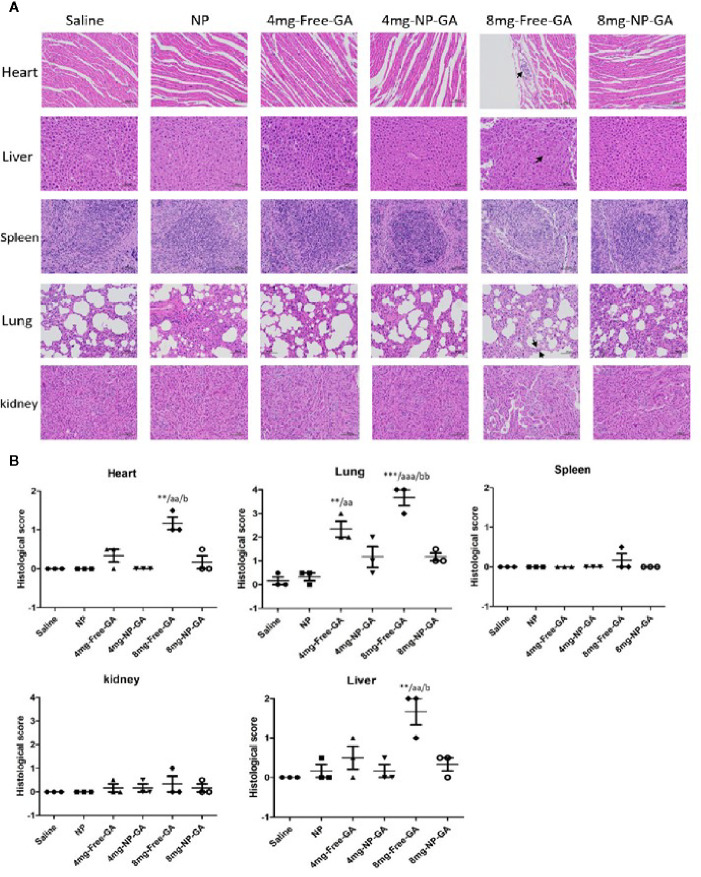
*In vivo* tissues and organ toxicity after free GA or GA-loaded FA-Arg-PEUU-NP (NP-GA) treatments. **(A)** H&E staining images and **(B)** quantification analysis of the heart, liver, spleen, lung, and kidney of the HCC1806-bearing xenograft mouse models after saline, FA-Arg-PEUU-NP (NP), free GA, or GA-loaded FA-Arg-PEUU-NP (NP-GA) treatments. The data are shown as the means ± SEM, n = 3 mice in each group. **p < 0.01, ***p < 0.001 compared to saline group; aa < 0.01, aaa < 0.01 compared to NP group; b < 0.05, bb < 0.01 compared to 8mg-NP-GA group.

H&E staining of the major organs was done to further suggest NP-GA does less damage to the off-target major organs in mice when compared to free GA. **Figures 7A, B** showed that NP-GA had less damage to the heart, liver, and lung than the free GA. These off-target H&E image data, (**Figure 7A**) along with their quantified scores (**Figure 7B**), are consistent with the lower levels of GA in these organs after NP-GA treatments (**Figure 6**). Neither NP-GA nor free GA caused significant damage to kidney and spleen (**Figures 7A, B**), although GA was detected in these organs (**Figures 6B–D**). Both the qualitative H&E stained image data and their quantified scores clearly illustrate the benefits of treating TNBC with NP-GA, that is, achieving higher anti-TNBC effects but with fewer off-target side effects to the major organs, particularly at the 8mg kg^-1^ GA dosage. Body weight variation is one of the parameters in toxicity testing.^34^ **Supplementary Figure S3A** showed the body weight of the mice in different groups during the course of the treatments, which had no apparent difference between groups. Percentages of the changes in body weight were also calculated, that is the % difference of the body weight of each mouse between day 6 and day 17. The results showed that NP-GA at both dosages did not create significant differences in the % changes in body weight when compared with saline and NP-alone groups; however, the 8mg-Free-GA group had body weight reduced and the % changes in body weight was significantly different from those in the saline and NP-alone groups (**Supplementary Figure S3B**). These data suggest that free GA treatment at a high concentration has toxicity to the mice, whereas the toxicity is not observed in the NP-GA treatment group.

### The FA-Arg-PEUU-NP Carriers Affect the Polarization of Tumor-Associated Macrophages (TAM)

Since Arg is present in the FA-Arg-PEUU NP carriers, it is interesting to examine whether the carrier *per se* or the GA-loaded NP will affect the polarization of TAM. Macrophages uptake Arg and process it *via* 2 different pathways: iNOS and arginase. The iNOS pathway produces M1 macrophage phenotype, while the arginase route produces M2 macrophage phenotype (33).

**Figure 8:**
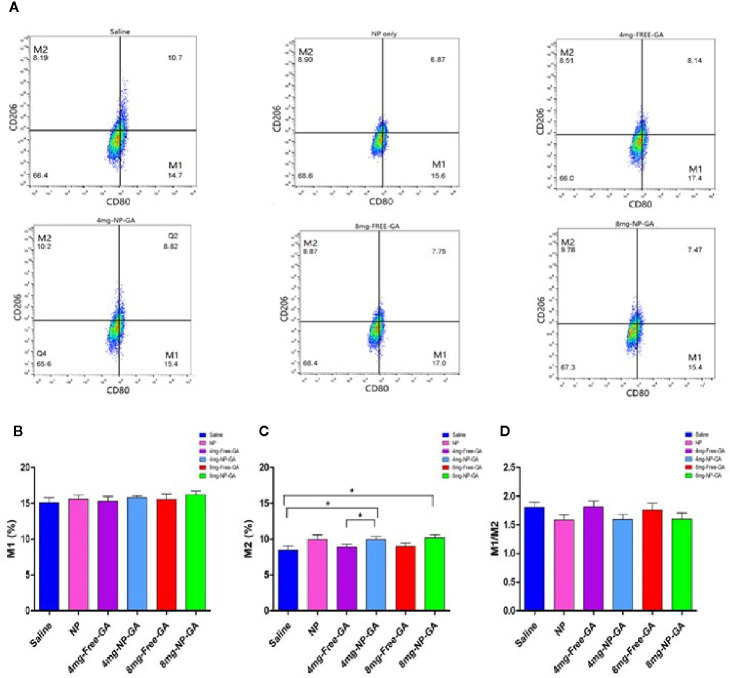
Polarization of tumor-associated macrophage (TAM) of the HCC1806-bearing xenograft mouse models after saline, FA-Arg-PEUU-NP (NP), free GA, or GA-loaded FA-Arg-PEUU-NP (NP-GA) treatments. **(A)** The polarization of the tumor-associated macrophages (TAM) of the HCC1806-bearing xenograft mouse models after the saline, FA-Arg-PEUU-NP (NP), free GA, or GA-loaded FA-Arg-PEUU-NP (NP-GA) treatments. The percentages of the **(B)** M1 phenotype, **(C)** M2 phenotype, and **(D)** M1/M2 ratio of the TAM. The data are shown as the means ± SEM, n = 5 mice in each group. *p < 0.05 as indicated.

**Figures 8A–C** showed that the FA-Arg-PEUU-NP *per se* neither affected M1 nor M2 population. The percentage of M1 after FA-Arg-PEUU-NP treatment was 15.61%, and was 15.10% in the saline group (**Figure 8B**), and the percentage of M2 after FA-Arg-PEUU-NP treatment was 9.95%, and was 8.45% in the saline group (**Figure 8C**).

When comparing between the NP-GA and saline, the NP-GA at both dosages had significantly higher M2 populations than the saline control (**Figure 8C**). The percentage of M2 in the saline group was 8.45% while it was 9.94% in the 4mg-NP-GA group and 10.17% in the 8mg-NP-GA group. When comparing between the NP-GA and free GA, NP-GA treatment resulted in a slightly higher average M2 population than free GA at the 4 mg kg^-1^ GA dosage level (**Figure 8C**). The M2 population of the free GA group was 8.89% and was 9.94% in the NP-GA group; the difference was statistically significance at p<0.05. However, NP-GA did not significantly affect the M2 population at the 8mg kg^-1^GA dosage level or the M1 population at both dosage levels (**Figures 8A–C**).

Although there was no statistical difference of TAM M1/M2 ratios in all treatments, it appears that the average TAM M1/M2 values of the NP-GA treatment at both GA dosage levels were lower than the corresponding free GA treatment (**Figure 8D**). Similarly, the blank FA-Arg-PEUU NP treatment also showed a lower average M1/M2 ratio than the saline control (**Figure 8D**).

## Discussion

Both *in vitro* and *in vivo* data in this study clearly show that the newly designed FA-Arg-PEUU-NP nano-carriers significantly increase the uptake of GA into TNBC, induce more apoptosis by activating both intrinsic and extrinsic apoptotic pathways, and exhibit more potent anti-TNBC effects with reduced off-target side effects. Our data strongly suggest that this new family of drug-loaded FA-Arg-PEUU-NP strategy can provide enhanced anti-TNBC effects regardless of the TP53 gene mutation status of the cancer. More importantly, the targeted delivery of GA to TNBC also significantly reduces off-target damage to the major organs, including the heart, liver, and lung, as demonstrated in the xenograft mouse models.

The efficacy and safety of GA for cancer treatment have been tested in clinical trials in China. In 2013, a phase IIa clinical study was conducted in China, which aimed to evaluate the safety and efficacy of GA treatment in patients with lung, gastrointestinal, breast, and liver cancer. Twenty-one patients received 45mg m^-2^ GA intravenously from days 1 to 5 for a 2-week cycle. The clinical outcomes showed that the objective response rate which indicates the proportion of patients with reduction in tumor burden was 14.3%, and disease control rate, which is a composite of overall response rate and stable disease, was 76.2%. The observed toxicity was mostly mild or moderate adverse events (AE) (Grades I and II), but not severe or life-threatening AE (Grade III and IV) based on a standard criterion (NCI-CTCAE 3.0) of anticancer drug toxicity evaluation. The symptoms of the AE were abdominal pain, injection site reactions, phlebitis, and nausea. The phase IIa clinical trial suggests that GA has a favorable safety profile and is effective in treating malignant cancers (17). However, a phase IIb clinical trial with 210 cases of non-small cell lung cancer (NSCLC), renal cell cancer, and colon cancer, and phase III clinical trial with 600 cases of advanced renal cell cancer and 300 cases of NSCLC documented that GA did not have an apparent advantage over chemotherapy, although no Grade III and IV adverse events were reported (Study report 138997640-2008ZX09101024/01 by Zhangleilei Jiangsu Kanion Pharmaceutical Company and Zhaoyiwu Jiangsu Kanion Pharmaceutical Company). The unsatisfactory clinical outcome of the free GA treatments in the malignant cancers led us to design FA-Arg-PEUU NP as a target nano-carrier for GA for the TNBC treatment, which can enhance therapeutic efficacy and also reduce off-target side effects as the data from this study showed. In the above clinical studies, the dosage of GA is at 45mg/m^2^, which corresponds to 1.215mg kg^-1^ assuming the reference body weight is 60kg and body surface area is 1.62m^2^. The equivalent mouse dose to that clinical GA dosage is 14.985mg kg^-1^, which is much higher than the dosages 4 or 8mg kg^-1^ of GA or GA-loaded FA-Arg-PEUU NP used in our current animal study. As shown in **Figure 4**, NP-GA and free GA treatments at these low GA dosage levels show significantly reduced tumor growth when compared to saline control, suggesting a dosage lower than the reported clinical trial dosage can be used to treat TNBC. Furthermore, at this low GA dosage, the treatment with the NP-GA strategy shows an even more significant reduction in tumor growth than the free GA treatment.

The targeted delivery of GA to TNBC is far less studied. The reported studies mainly use NP carriers to deliver GA to non-TNBC breast cancer (34–36). For example, a study used MDA-MB-231 cells, MCF-7 breast cancer cell lines, and 4T1 cell-bearing mice as models to study the anti-cancer effect of the co-delivery of GA and TNF-related apoptosis-inducing ligand (TRAIL) plasmid by hyaluronic acid grafted PEI-PLGA nanoparticles (GA/pTRAIL-HA/PPNPs) (37). It is known that TRAIL *per se* induces apoptosis by binding to the TRAIL death receptors, which leads to the recruitment of Fas-associated death receptor and triggers the consequent apoptotic signaling cascade. The combined delivery of GA/pTRAIL-HA/PPNPs shows an enhanced anti-cancer effect over GA-HA/PPNPS and pTRIAL-NA/PPNPs as demonstrated in 4T1 cell-bearing mice. The tumor suppression rate of GA-HA/PPNPs, TRAIL-HA/PPNPs, and GA/pTRAIL-HA/PPNPs are 46.4%, 62.0%, and 84.1%, respectively. The enhanced anti-cancer effect of GA/pTRAIL-HA/PPNPs is due to the increased apoptosis level in the breast cancer cells. Co-delivery of TRAIL with GA demonstrates a pragmatic strategy which may be beneficial to modify our current FA-targeted Arg-PEUU-NP delivery system to further enhance the apoptosis level in the tumor sites. However, this study did not use free GA as reference group and hence the anti-cancer effects between free GA and GA/pTRAIL-HA/PPNPs cannot be assessed. However, our current study design permits such an assessment to determine whether GA delivered by the FA-Arg-PEUU-NP treatment would be better than free GA treatment.

The current data also show that NP-GA does significantly less damage to the heart, lung, and liver in the TNBC-bearing mouse model when compared to free GA (**Figure 7**). Therefore, the data in this study provide strong evidence to suggest a relatively low dosage of NP-GA can be used to treat TNBC with fewer off-target side effects. The reason behind this observed lower off-target side effect in the NP-GA treatment is attributed to the targeted delivery of GA to TNBC that highly expresses folate receptors (FRα) (30). FRα have a highly restricted expression in the luminal membrane of secretory ductal cells in normal breast [40]. FRα are also not expressed or have little expressions in the peritoneum, colon, small intestine, kidney, and ovary (38, 39). The specific expression of FRα in tumor has led to the development of FRα-targeted therapies which are in clinical trials for the treatment of TNBC. A multi-epitope FRα peptide vaccine is undergoing a randomized phase II trial studies with TNBC patients in the Academic and Community Cancer Research United in the US (ClinicalTrials.gov Identifier: NCT03012100).

The current target therapies for TNBC, such as epidermal growth factor receptor-targeted approach with cetuximab, only demonstrates a modest anti-cancer activity in the metastatic TNBC (40); bevacizumab, which targets vascular endothelial growth factor, does not show a pronounced benefit of overall survival (41). Concerning these unsatisfactory therapeutic outcomes, our proposed FRα-targeted NP-GA therapeutic strategy appears to be a better alternative to the TNBC treatment.

In this study, our data also show interesting and useful information about TNBC that harbor TP53 mutations. The NP-GA treatments induce apoptosis in TNBC in a p53-independenet manner. The TP53 gene is mutated in approximately 80% of TNBC cases (42, 43). Mutations in TP53 in TNBC are predominantly missense mutations, producing mutant p53 proteins that promote tumorigenesis (42). Our data demonstrate that NP-GA exerts a potent therapeutic effect in TNBC which either harbor TP53 mutations or has no TP53 expression. The enhanced apoptosis level after NP-GA treatment is, at least in part, due to the enhanced intrinsic and extrinsic apoptosis *via* the activation of caspases, but not the collapse of mitochondrial membrane potential. In general, the caspase activation is preceded by opening of the mitochondrial permeability transition pore (MPT), followed by dissipation of Δψm and release of cytochrome c. A possible explanation of our results is that mitochondria can also transiently maintain a membrane potential after the release of the apoptosis-inducing factors (44). Alternatively, NP-GA may induce a different regulation on the apoptotic machinery in TNBC, in which MPT may not be required for mitochondrial outer membrane permeabilization or cytochrome c release, and MPT may not participate in the apoptosis (45). Indeed, induction of caspase-3 activity and apoptosis in human blood granulocytes occurs without prior mitochondrial changes, such as the loss of mitochondrial membrane potential and release of cytochrome c (46). Further study is needed to examine the role of Δψm in GA-induced apoptosis in TNBC.

In addition to a conventional mechanistic study to reveal the mechanism of action underlying the enhanced therapeutic effects of NP-GA, TAM level was also assessed to determine whether the newly designed FA-Arg-PEUU-NP drug nano-carrier would have any effect on TAM. In the tumors of the TNBC patients, TAM has the highest abundance and accounts for 25% of the total cell population (47). The increased recruitment of TAM to TNBC may be due to the reduced expression of Raf kinase inhibitory protein and reduced secretion of chemokines CCL5 in TNBC (48). The large infiltrate of TAM is associated with a high risk of distant metastasis, low disease-free survival, and low overall survival in the TNBC patient (49).

In this study, only a pure Arg was used as the amino acid building block in the FA-tagged Arg-PEUU-NP for the delivery of GA for TNBC treatment. Our *in vivo* TAM data show that the blank FA-Arg-PEUU-NP and the GA-loaded FA-Arg-PEUU-NP do not affect the M1 population (**Figure 8B**), but slightly increase (statistically significant at p<0.05) the M2 population in the TNBC tumors (**Figure 8C**), and hence cause a decrease in the M1/M2 ratio (**Figure 8D**). The incorporation of GA in the FA-Arg-PEUU-NP neither changes the M2 population nor the M1/M2 ratio against the blank FA-Arg-PEUU-NP, suggesting GA *per se* does not affect the polarization of the macrophages. Although M2 macrophages are known to promote cancer progression, the TNBC tumor size and tumor weight data (**Figure 4**) show that blank FA-Arg-PEUU-NP *per se* does not affect tumor growth *in vivo* when compared to the saline control, suggesting that the slight increase in the M2-polarized macrophage population in the tumor induced by the Arg-based nanoparticle carrier (Arg-PEUU-NP) does not promote the TNBC tumor growth. Our data suggest the extreme complexity of the role of TAM, and the role of Arg in TAM polarization and its effect on tumor growth. Therefore, additional in-depth study of the role of TAM under our GA-loaded FA-Arg-PEUU-NP strategy is needed to clarify the impact of the Arg-based NP delivery vehicle on TAM polarization. It is important to consider all the factors involved in assessing the collective effect of a new strategy on cancer therapy.

Since TAM changes the tumor microenvironment that promotes cancer development and progression, targeting TAM may be a potential therapeutic strategy to treat cancers. For example, the mannose receptors that are overexpressed in TAM can be used as a target for the design of nanoparticles for drug delivery. Previously, poly(lactic-co-glycolic)acid (PLGA) nanoparticles that are PEGylated with acid sensitive sheddable polyethylene glycol (PEG) and surface-modified with mannose (i.e. AS-M-PLGA-NPs) have been designed to actively target the nanoparticles to TAMs *via* mannose-mannose receptor recognition after acid-sensitive “shedding” of the PEG in the relatively low pH tumor microenvironment (50). The use of these nanoparticles to carry doxorubicin, i.e., TAM-targeting DOX-AS-M-PLGA-NPs, demonstrates a more potent effect than DOX alone or DOX-AS-PLGA-NPs in controlling the tumor growth of the orthotopic MMTV-M-Wnt-1 mammary tumor-bearing mouse model (50). A similar design can be incorporated into our GA-loaded FA-Arg-PEUU-NP to further enhance its therapeutic effects in TNBC treatment.

Another possible strategy to treat TNBC is to modulate the macrophage polarization. Arg is a common substrate for nitric oxide synthase and arginase pathways after macrophage uptake Arg. Depending on the type of Arg metabolic pathway, very different macrophage phenotypes can be produced. The M1 macrophage will convert Arg to citrulline and nitric oxide (NO), which is inflammatory and cytotoxic; the M2 macrophages hydrolyzes Arg to urea and ornithine, which is important for cellular proliferation and tissue repair (51, 52). Previously, the Chu lab has shown that the amino acid-based poly(ester amide) or poly(ester urea urethane) biomaterials synthesized from Arg have a tunable immune-modulating property (16, 24, 27, 28). For example, He et al. recently demonstrated that by incorporating an Arg derivative (L-nitroarginine, NOArg) into a pure Arg as the amino acid building block, a new family of NOArg-Arg PEA copolymers is synthesized that possesses the ability to polarize M1 to M2 and exhibits a wound healing property, as demonstrated in a diabetic 3^rd^ degree burn wound mouse model (28). The significantly faster and better wound healing quality in the burn wound diabetic model is achieved with an optimal balance of M1/M2 mixture population more toward M2. Such a preferred M1 to M2 mixture population can only be achieved with a proper NOArg to pure Arg ratio in the NOArg-Arg PEA copolymers. More importantly, neither a pure NOArg nor pure Arg-based PEA can achieve such a preferred M1 to M2 mixture population for the desirable and more efficient wound healing quality. For the treatment of TNBC, a combination of NP-GA and Th1 cytokine interferon-γ (IFN-γ) can be administrated to enhance M1 polarization in the TNBC in our future study. IFN-γ is a useful adjuvant immunotherapy for many cancers (53). It also inhibits angiogenesis in the tumor (53).

In addition, the use of Arg-based nanoparticles as the carriers for anticancer drugs like GA for TNBC treatment may have the advantage of making the surface of the nanoparticles positively charged. It is reported that the dominant mechanism for the uptake of nanoparticles is an active process instead of a passive diffusion (54). Such an active uptake of cationic NPs like Arg-PEUU-NP (+10mV charge) (16) by cancer cells is further enhanced by the general anionic surface membrane of cells, particularly cancer cells, that express excessive anionic-charged sialic acid (55–60). Holmberg et al. (55) and Gakhar et al. (61) reported that the growth of tumor cells, including PC3 prostate cancer cells, is inhibited by the cationic polymers, such as dextran derivatives and vinyl-based Poly(AETA) synthesized from the [2-(acryloxy)ethyl]trimethylammonium chloride] monomer (AETA). Their data suggest that certain cationic polymers may represent an alternative strategy to target and inhibit the growth of malignant cancers.

Therefore, as shown in our current TNBC study, the design of cationic folate-targeted Arg-based pseudo-protein nanoparticles as anticancer herbal compound carriers offer many beneficial effects like overcoming the anionic cell membrane barrier, promoting internalization, and facilitating tumor-targeting for a more efficient delivery of the drug. Therefore, the design achieves better therapeutic efficacy with reduced off-target side effects when compared to free drug delivery.

## Conclusion

In this study, a new targeted therapy for TNBC with a Chinese medicinal compound gambogic acid (GA) delivered by a newly designed folate (FA)-tagged biodegradable amino acid-based poly(ester urea urethane) nanoparticles, the GA-loaded FA-Arg-PEUUs (NP-GA), is reported. The conjugation of FA in the Arg-PEUU nano-carriers increases the cellular uptake of the nano-carriers into the TNBC both *in vitro* and *in vivo*. Compared to free GA, the NP-GA treatments exert higher cytotoxicity with increased intrinsic and extrinsic apoptosis in TNBC cells that either harbor TP53 mutations or without p53 protein expression. In the TNBC-bearing xenograft mouse model, the NP-GA treatments also demonstrate better therapeutic efficacy with increased apoptosis in the tumors and reduced off-target damages, as there is more efficient targeted delivery of GA to the tumor sites instead of non-discriminated delivery to other organs. Further investigations may develop this NP-GA system as alternative therapeutics for TNBC treatment.

## Data Availability Statement

The raw data supporting the conclusions of this article will be made available by the authors, without undue reservation.

## Ethics Statement

The animal study was reviewed and approved by Hong Kong Baptist University.

## Author Contributions

Conceptualization: C-CC, ZB, HK, and QX. Data curation and formal analysis: QX and RG. Funding acquisition: C-CC and ZB. Writing: HK and QX. Review and editing: C-CC and ZB. All authors contributed to the article and approved the submitted version.

## Funding

This study is supported by The Vincent and Lily Woo Foundation to both Chu at Cornell University in the USA and Bian in Hong Kong Baptist University in Hong Kong. The supported project title is “East Meet West: A revolutionized nanotechnology approach to modernize the delivery of Chinese medicine for vast improved cancer therapeutic efficacy”.

## Conflict of Interest

The authors declare that the research was conducted in the absence of any commercial or financial relationships that could be construed as a potential conflict of interest.

## Supplementary Material

Supplementary Figure 1 The viability of **(A)** HCC1143 and **(B)** HCC1806 cells after free GA or GA-loaded FA-Arg-PEUU-NP (NP-GA) treatments for 24 hours at the indicated concentrations. The data are shown as the means ± SEM, n = 3.

Supplementary Figure 2 The apoptotic TNBC cell distribution after free-GA or GA-loaded FA-Arg-PEUU-NP (NP-GA) treatments for 3 hours.

Supplementary Figure 3
**(A)** Body weight and **(B)** percentage changes in body weight of the HCC1806-bearing xenograft mouse models during the treatment period. The data are shown as the means ± SEM, n = 4-5 mice in each group. *p<0.05 compared to saline group; a<0.05 compared to NP group.
