# Supplementary figures and images for: Targeted Chinese Medicine Delivery by A New Family of Biodegradable Pseudo-Protein Nanoparticles for Treating Triple-Negative Breast Cancer: In Vitro and In Vivo Study
Source: Front Oncol. 2021 Jan 20;10:600298. doi: 10.3389/fonc.2020.600298 (PMC7855979; doi:10.3389/fonc.2020.600298)

**a**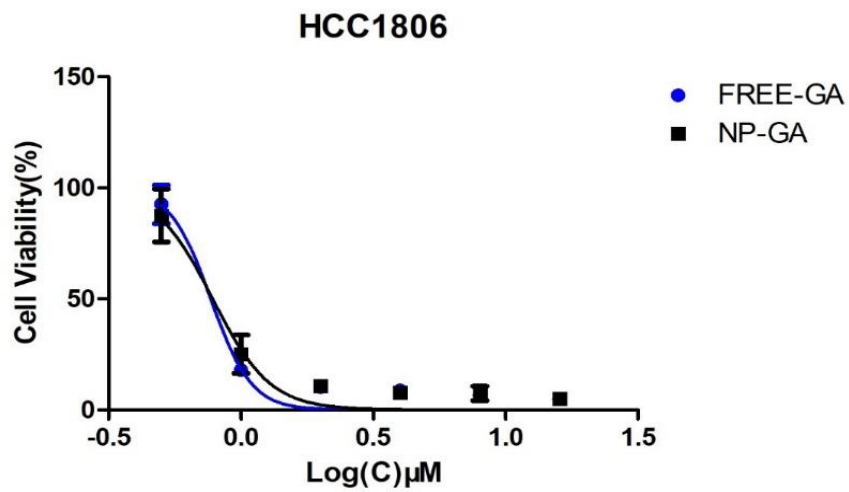**b**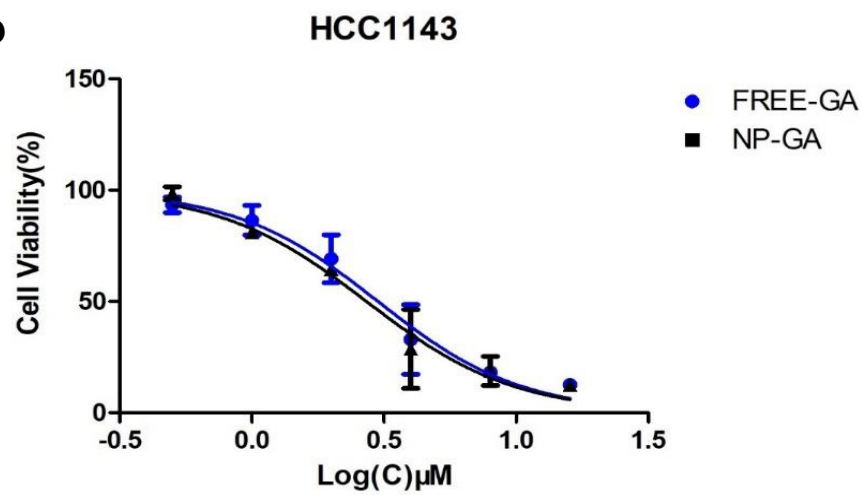

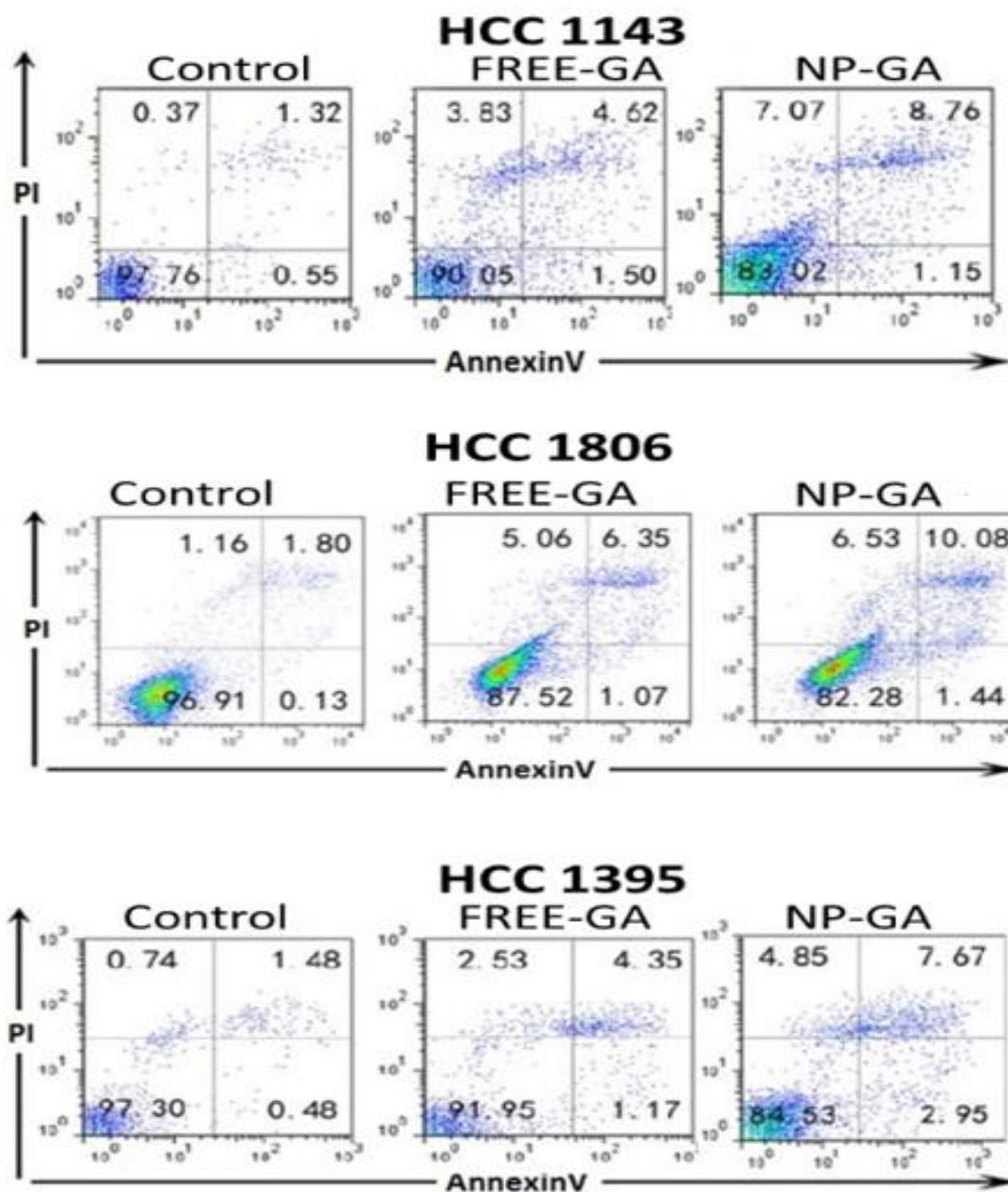

**a**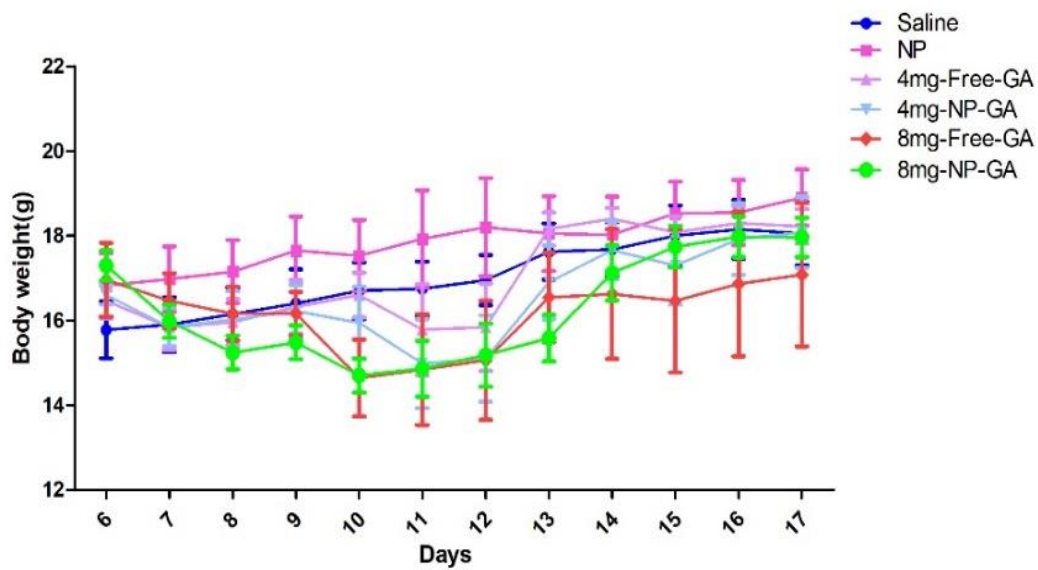**b**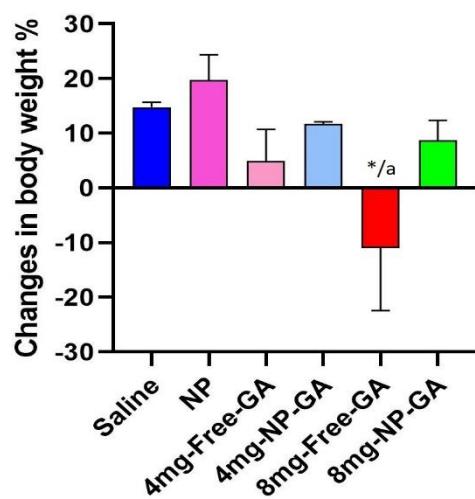

Supplement: Supplementary Figure 1 — The viability of (A) HCC1143 and (B) HCC1806 cells after free GA or GA-loaded FA-Arg-PEUU-NP (NP-GA) treatments for 24 hours at the indicated concentrations. The data are shown as the means ± SEM, n = 3. [file DataSheet_1.pdf]
